# Comparison of Fermentation and Wines Produced by Inoculation of *Hanseniaspora vineae* and *Saccharomyces cerevisiae*

**DOI:** 10.3389/fmicb.2016.00338

**Published:** 2016-03-16

**Authors:** Jessica Lleixà, Valentina Martín, María del C. Portillo, Francisco Carrau, Gemma Beltran, Albert Mas

**Affiliations:** ^1^Departament Bioquímica i Biotecnologia, Facultat d'Enologia, Universitat Rovira i VirgiliTarragona, Spain; ^2^Sección Enología, Food Science and Technology Department, Facultad de Química, Universidad de la RepúblicaMontevideo, Uruguay

**Keywords:** non-*Saccharomyces*, *Hanseniaspora*, alcoholic fermentation, PCR-DGGE, massive sequencing

## Abstract

Interest in the use of non-*Saccharomyces* yeasts in winemaking has been increasing due to their positive contributions to wine quality. The non-*Saccharomyces* yeast *Hanseniaspora vineae* is an apiculate yeast that has been associated with the production of wine with good aromatic properties. However, little is known about the fermentation dynamics of *H. vineae* in natural must and its interaction with autochthonous yeasts. In the present study, we performed semi industrial fermentations of Macabeo and Merlot musts inoculated with either *H. vineae* or *S. cerevisiae*. The yeast population dynamics were monitored by plate culturing, PCR-DGGE and massive sequencing techniques. The results obtained with these techniques show that *H. vineae* was able dominate the autochthonous microbiota in Macabeo must but not in Merlot must, which exhibited a larger, more diverse yeast population. The presence of *H. vineae* throughout most of the Macabeo fermentation resulted in more fruity and flowery wine, as indicated by the chemical analysis of the final wines, which demonstrated a strong presence of phenyl ethyl acetate at concentrations higher than the threshold of perception and approximately 50 times more than that produced in wines fermented with *S. cerevisiae*. This compound is associated with fruity, floral and honey aromas.

## Introduction

Wine is the result of the alcoholic fermentation of grape must. Alcoholic fermentation is driven by yeasts, and it consists of the transformation of sugars present in the must, glucose and fructose, into ethanol and carbon dioxide. The yeast species *Saccharomyces cerevisiae* is considered to be primarily responsible for this process. *S. cerevisiae* is known for only metabolizing sugars via the fermentative pathway when the sugar concentration is high, even in the presence of oxygen. This phenomenon is known as the Cabtree effect (Cabtree, 1929).

Winemaking is currently changing because of an emerging interest in the use of non-*Saccharomyces* yeasts during alcoholic fermentation to increase wine complexity and differentiation. Non-*Saccharomyces* yeasts are commonly found on the grape surfaces, and these yeasts have been associated with spontaneous and unpredictable fermentation, which can result in arrested or sluggish fermentation and wine spoilage. Nevertheless, several recent studies have shown that these yeasts positively affect wine fermentation and the final wine. The positive role of non-*Saccharomyces* ranges from a better fermentation performance to improve wine quality and complexity (Fleet, 2008; Jolly et al., 2014).

Non-*Saccharomyces* yeasts can contribute to the sensorial profile of wine as a result of the production of various metabolites and the activity of certain enzymes that interact with the precursors of aromatic compounds, such as β-glucosidases, which are present in many non-*Saccharomyces* yeast but not in *S. cerevisiae*. β-glucosidases hydrolyze aromatic glycosylated precursors into free volatile compounds to improve the final wine flavor (Swangkeaw et al., 2011; Jolly et al., 2014). Many other enzymes of technological relevance are also secreted by non-*Saccharomyces* yeasts, such as pectinases. Enzymes with proteolytic activity are of key interest in enological fields because they facilitate the clarification process in wine and improve protein stability (Strauss et al., 2001; Maturano et al., 2012).

These yeasts have garnered interest in winemaking due to their beneficial effects and because consumers are demanding new wine styles. Many commercial yeast companies have also begun to promote mixed and sequential wine fermentations in order to satisfy consumer and producer demands. Therefore, companies have begun to thoroughly study and commercialize non-*Saccharomyces* strains, like *Torulaspora delbrueckii* or *Metschnikowia pulcherrima* (Jolly et al., 2014). Moreover, some of the yeast species that are being evaluated belong to *Hanseniaspora* spp., the main non-*Saccharomyces* yeasts in grape must that are considered apiculate yeasts due their cell morphology. Specifically, the yeast *Hanseniaspora vineae* (anamorph *Kloeckera africana*) of this genus has been of great interest because it produces several key aromatic compounds (Viana et al., 2011; Medina et al., 2013).

The strain of *H. vineae* used in this study was isolated from Uruguayan vineyards and selected due to its positive effect on wine fermentation and good contribution to the aroma profile of the final wine. *H. vineae* has been demonstrated to increase fruity aromas and produce a high amount of acetate esters, such as 2-phenylethyl acetate and ethyl acetate, in wines elaborated by sequential fermentation with *S. cerevisiae* (Viana et al., 2011; Medina et al., 2013).

In summary, the use of non-*Saccharomyces* yeasts to produce new wine styles has been increasing due to the different aromatic profiles obtained. The aim of this work was to compare the fermentation dynamics of *H. vineae* and *S. cerevisiae* and the different obtained wines after the inoculation of these two species. To this end, we used natural must from two grape varieties, Macabeo and Merlot, inoculated either with *H. vineae* or *S. cerevisiae* fermented in semi-industrial conditions. The yeast population dynamics were monitored by plate culturing, PCR-DGGE and 18S rRNA gene massive sequencing techniques. To confirm the differences between the two species, the final wines underwent a sensory evaluation, and the aromatic profile was determined.

## Materials and methods

### Yeast strains

The commercial wine yeast strain used in this study was *Saccharomyces cerevisiae* QA23 (Lallemand®). The apiculate yeast strain used in this work, *H. vineae* T02/5AF, was isolated from Uruguayan vineyards. Strain QA23 of *S. cerevisiae* was obtained in active dry yeast (ADY) form and rehydrated according to the manufacturer's instructions (Lallemand®). The *H. vineae* strain T02/5AF was obtained in fresh paste form and rehydrated in the same manner as QA23 using warm water. The inoculation was in both cases 2 × 10^6^ cells/ml of must.

### Fermentation conditions

The Macabeo and Merlot grape varieties were fermented at the experimental cellar of the Faculty of Enology (Mas dels Frares, Tarragona Spain). The Macabeo musts were fermented in triplicate in 100 l tanks at 18°C, and 6 kg of Merlot grapes were fermented in 8 l submerged cap fermentation tanks at 26°C. The Macabeo must was submitted to a vacuum filtration process, whereas the Merlot grapes were selectively handpicked in the vineyard.

Fermentation activity was followed by daily density monitoring using a portable densimeter (Mettler Toledo). Samples were taken once a day from each fermenter and studied as described in the following sections.

### Cell growth measurements

Samples were taken once a day, diluted in sterile MilliQ water (Millipore Q-POD™ Advantage A10), plated on YPD medium (Glucose 2%, Peptone 2%, Yeast Extract 1%, Agar 1.7%) and lysine agar medium (Oxoid, England) plates using an automated spiral platter WASP II (Don Whitley. Scientific Limited, England), and incubated at 28°C for 48 h. The YPD medium provided the total yeast counts, whereas the lysine agar medium only provided the non-*Saccharomyces* cell counts because *S. cerevisiae* cannot grow using lysine as a unique nitrogen source. Appropriate dilution plates were counted, and 20 colonies from the must before the inoculation and the beginning (density 1070 for Macabeo and 1090 for Merlot, both of them at day 1), middle (density between 1050 and 1040) and end (density below 1000, and residual sugars below 5 g/l) of the fermentation were randomly selected and purified on YPD plates for yeast identification.

### Yeast identification

The yeasts were identified based on the RFLPs of the PCR-amplified ITS-5,8S rDNA region from the isolated colonies as described by Esteve-Zarzoso et al. (1999). The RFLP patterns of the yeast isolates were compared with those of the www.yeast-id.org (https://www.yeast-id.org/) based on the method described by Esteve-Zarzoso et al. (1999) and grouped to a known yeast species. Yeast identification was confirmed by sequencing the amplified D1/D2 domain of the 26S rDNA of representative colonies of each identified group as described by Kurtzman and Robnett (1998) and comparing this sequence with those of the type strains included in GenBank®. Identification was considered appropriate with similarities higher than 99%. The sequencing was performed by Macrogen.

*Saccharomyces cerevisiae* cells from the isolated colonies identified as *S. cerevisiae* were further characterized by Interdelta PCR analysis as described by Legras and Karst (2003).

### Massive sequencing analysis

DNA (5-100 ng) was extracted from 1 ml samples taken at the beginning, middle and end of the fermentation using the recommended procedure for the DNeasy Plant Mini kit (Qiagen, Hilden, Germany), including three bead-beating steps for 3 min in a FastPrep-24 bead beater (MP Bio, Solon, OH) to homogenize the samples. The extracted DNA was stored at −20°C until further processing. A 350 bp (on average) 18S rRNA gene fragment was amplified in triplicate from each DNA sample with the universal primers FR1 (5-ANCCATTCAATCGGTANT-3) and FF390 (5-CGATAACGAACGAGACCT-3) (Chemidlin Prévost-Bouré et al., 2011). All primers had an Ion Torrent tag, and the universal primer included a 10-bp barcode unique to each amplified sample. The PCR reactions contained 5–100 ng DNA template, 1 × GoTaq Green Master Mix (Promega), 1 mM MgCl_2_, and 2 pmol of each primer. The reaction conditions consisted of 94°C for 3 min, followed by 35 cycles of 1 min at 94°C, 1 min at 52°C and 1 min at 72°C, and a final extension phase for 10 min at 72°C. The PCR products were pooled by sample and cleaned using a GeneRead Size Selection kit (Qiagen, Hilden, Germany). The cleaned PCR products were submitted to the Centre for Omic Sciences (Reus, Spain), where their quality was assessed with a Bionalyzer and their quantity was adjusted for sequencing. The raw sequences were demultiplexed and quality filtered using QIIME v1.8.0 (Caporaso et al., 2010a). Reads were discarded if the average quality score of the read was <25, if the length of the read was <200 or >400 and they contained one or more ambiguous base calls. Operational taxonomic units (OTUs) were assigned using QIIME's uclust-based (Edgar, 2010) open-reference OTU-picking workflow with a threshold of 97% pairwise identity. The OTU sequences were aligned using PYNAST (Caporaso et al., 2010b) against the SILVA 119 reference database (Pruesse et al., 2007). Taxonomic assignments were made in QIIME against the SILVA 119 database using the naive Bayesian classifier rdp (Wang et al., 2007). The template alignment of the Greengenes core set filtered at 97% similarity. The OTU taxonomy was determined using the RDP classifier retrained toward the GreenGenes bacterial 16S rRNA database (13_8 release) (DeSantis et al., 2006) at 97% similarity. Chimeric sequences were identified and removed using ChimeraSlayer (Haas et al., 2011), and a phylogenic tree was generated from the filtered alignment using FastTree (Price et al., 2009). A final OTU table was created that excluded excluding unaligned sequences and singletons (sequences observed just once). To avoid biases generated by differences in sequencing depth, the OTU table was rarified to an even depth of 20,000 sequences per sample in comparisons of all sample types in this study.

### PCR-DGGE

The U1GC/U2 primers were used to amplify the specific U1/U2 domain of the 28S ribosomal region of yeast (Meroth et al., 2003). The PCR amplifications were performed on a Gene Amp PCR System 2700 (Applied Biosystems, Fosters City, USA) using EcoTaq DNA Polymerase (Ecogen, Spain). The Dcode universal mutation detection system (Bio-Rad, Hercules, Calif.) was used to run the DGGE analysis. The amplification of the fragments and denaturing electrophoresis were performed according to Meroth et al. (2003). The bands were excised from the gels, and the DNA was eluted overnight in 40 μl of 10 mM Tris pH 8 and 1 mM EDTA (TE) at 4°C. The DNA was re-amplified with the same pair of primers without the GC-clamp and sequenced by Macrogen. The BLASTN algorithm was applied to the GenBank database to identify sequences (http://www.ncbi.nlm.nih.gov/BLAST/). We considered appropriate the identification of the sequences with the corresponding type strains sequences when the sequence identity was higher than 98%.

### Analysis of volatile compounds

The aromatic compounds were extracted using adsorption and separate elution from an isolute ENV+ cartridge packed with 1 g of highly crosslinked styrene-divinyl benzene (SDVB) polymer (40–140 mm, cod. no. 915- 0100-C), as previously reported by Boido et al. (2003). The cartridges were sequentially equilibrated with methanol (15 mL) and distilled water (20 mL). A sample of 50 mL of wine diluted with 50 mL of distilled water and containing 0.1 mL of internal standard (1-heptanol at 230 mg/L in a 50% hydroalcoholic solution) was applied with an appropriate syringe (4–5 mL/min), and the residue was washed with 15 mL of distilled water. The aromatic compounds were eluted with 30 mL of dichloromethane. The solution was dried with Na_2_SO_4_, concentrated to 1.5 mL on a Vigreux column, stored at 10°C, and, immediately prior to GC–MS analysis, further concentrated to 150 μL under a gentle nitrogen stream. The GC/MS analyses were conducted using a Shimadzu-QP 2010 ULTRA (Tokyo, Japan) mass spectrometer equipped with a Stabilwax (30 m × 0.25 mm i.d., 0.25 μm film thickness) (Restek) capillary column. The components of the wine aromatic compounds were identified comparing their linear retention indices with those of pure standards. (Aldrich, Milwaukee, 194 WI). The mass spectral fragmentation patterns were also compared with those stored in databases. GC-FID and GC-MS instrumental procedures using an internal standard (1-heptanol) were applied for quantitative purposes, as described previously by Boido et al. (2003). Ethanol and residual sugars were quantified using Winescan FT 120 (WineScan FT120 Type 77110, Foss Analytical, Denmark).

### Sensory analysis

A specialized panel (13 panelists) analyzed the sensorial attributes of Macabeo and Merlot wines fermented with *H. vineae* and *S. cerevisiae*. The wines were analyzed by means of a triangle test and descriptive analysis. The aim of the triangle test was to distinguish the wine fermented with *H. vineae* from the wine fermented with *S. cerevisiae*. The descriptive test emphasized the aroma and flavor attributes: Reduction, fresh fruit, candied fruit, flowery, aromatic plant, yeast, toasted (phenolic), herbaceous, aroma, sourness, structure, bitterness, volume and global impression.

### Statistical analysis

The variance the aromatic compounds was analyzed using the Statistica 7.1 software (StatSoft, Tulsa, OK, 1984-2005). The sensory analysis results were submitted to Student's *t*-test. The results were considered significant when the associated *p*-value was below 0.05.

## Results

### *H. vineae* and *S. cerevisiae* fermentations

**Figure 1:**
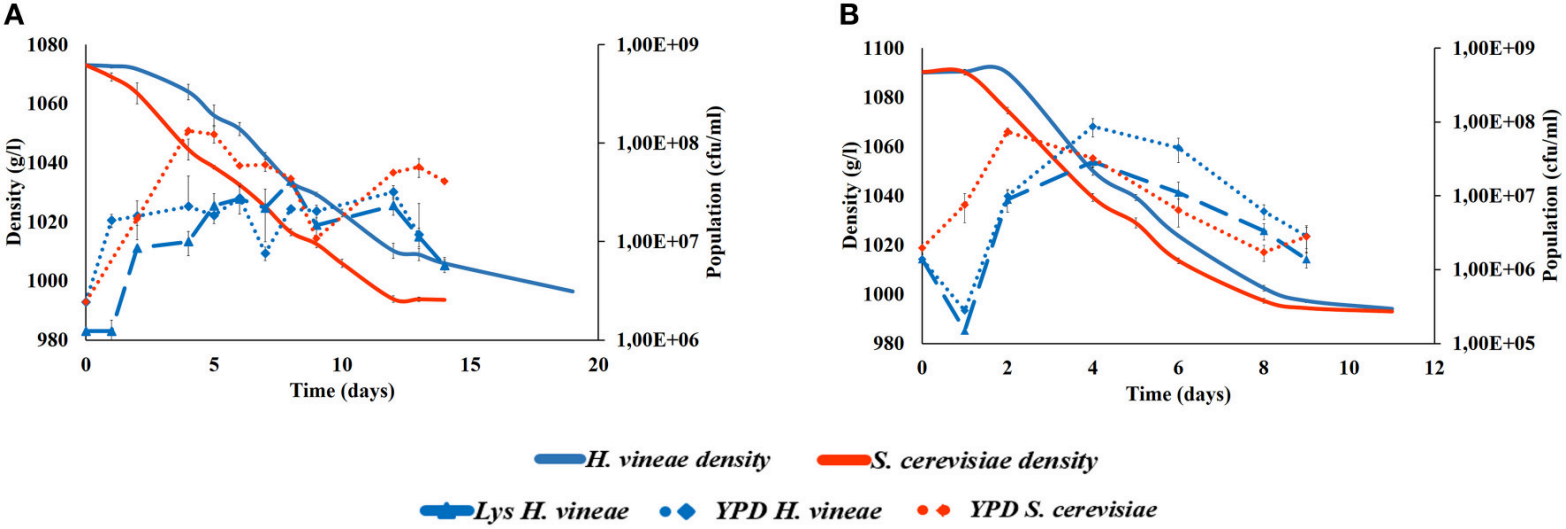
**Density measures of fermentations kinetics (−) and yeast population (cfu/ml) growth in YPD (♦) and lysine agar (▴) medium of Macabeo (A) and Merlot (B) tanks inoculated with ***H. vineae*** (Blue) or ***S***. ***cerevisiae*** (Red)**.

The changes in the density and yeast populations during the alcoholic fermentations of both Macabeo and Merlot grapes are presented in Figure 1. The Macabeo must (Figure 1A) inoculated with *H. vineae* required a longer fermentation process (19 days) than those inoculated with *S. cerevisiae* (14 days) due to slower fermentation kinetics and a longer latency phase. However, Merlot grapes (Figure 1B) inoculated with *H. vineae* and with *S. cerevisiae* showed a similar fermentative progress, completing the fermentation in 9 days. This fact could be explained by the early presence of non-inoculated *S. cerevisiae* in the first stages of the fermentation.

No significant differences were observed in the ethanol concentration obtained at the end of the fermentation of both varieties (10.75 ± 0.20 for Macabeo and 12.75 ± 0.10 for Merlot wines). Although all the wines were considered as “dry” (sugar concentration bellow 2 g residual sugars /L), a small difference was observed in the residual sugars in the Macabeo fermentation because the musts fermented with *H. vineae* left 1.7 ± 0.3 g fructose/L, while all the other wines each of the residual sugars (glucose or fructose) were below 1 g/L.

The yeast population was quantified based on the colony growth on YPD and lysine agar medium. The total yeast population (YPD) was similar for the Macabeo and Merlot fermentations. The non-*Saccharomyces* yeasts counts (lysine agar) were slightly lower than the total yeast population counts in tanks inoculated with *H. vineae* for both grape musts. The Macabeo must was submitted to a vacuum filtration, which reduced the initial yeast population and resulted in yeast counts of 8.8 × 10^4^ cfu/ml on YPD and 5.8 × 10^4^ on lysine agar in must before inoculation.

### Yeast biodiversity in merlot and macabeo musts

**Figure 2:**
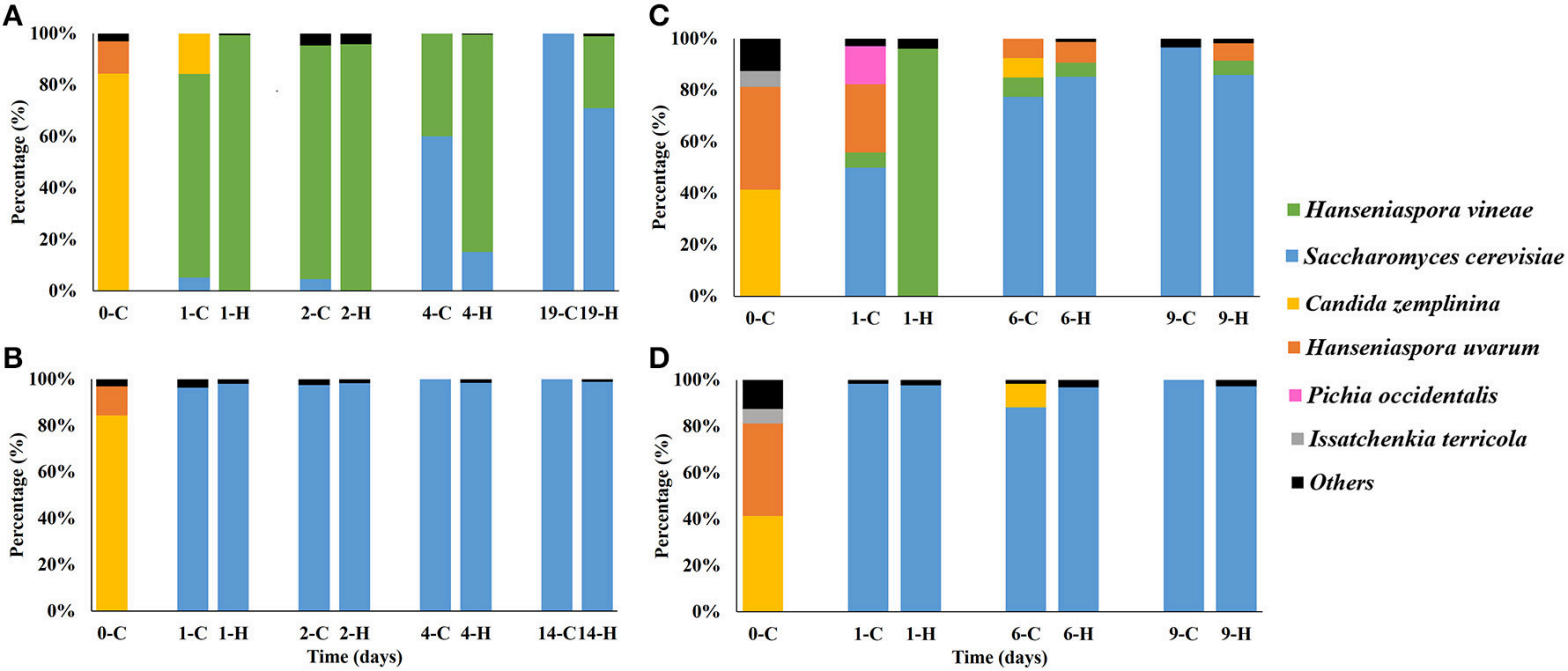
**Comparison of the percentage of yeast species grown in YPD medium and the main genera detected by HTS along the fermentation from Macabeo tanks inoculated with ***H***. ***vineae*** (A) or ***S. cerevisiae*** (B) and Merlot tanks inoculated with ***H. vineae*** (C) or ***S***. ***cerevisiae*** (D)**. The letters C (Culture) and H (HTS) correspond to the different techniques used. The results included as *others* refer to the yeast species that represent less than 5% of the total yeast population.

We identified only three yeast species in Macabeo must (Figures 2A,B), with *Candida zemplinina* being the main yeast species representing more than 80% of the yeast population. The other two yeast species identified were *Hanseniaspora uvarum* and *Torulaspora delbrueckii*. Of these, *H. uvarum* represented 12.50% of the total yeast population, whereas *T. delbrueckii* represented only 3.13% of the population. This distribution significantly differed in the yeast population recovered from Merlot must (Figures 2C,D). We identified up to eleven yeast species, with *C. zemplinina* and *H. uvarum* being the main species representing a percentage of 41 and 39% of the total yeast population, respectively. The low yeast diversity in Macabeo must may be due to the prefermentative filtration protocol, which reduces the yeast population. Moreover, during Merlot fermentation the must maintains contact with grape skins, which releases yeasts during the whole process. In both musts, only non-*Saccharomyces* yeasts were detected.

### Yeast population dynamics during fermentation

The yeast population dynamics during fermentation after the inoculation of *S. cerevisiae* or *H. vineae* was followed in yeast colonies grown on YPD plates based on PCR-DGGE, and the massive sequencing of the 18S rRNA gene at the beginning, middle and end of the fermentation.

For the Macabeo fermentation, the inoculated strain accounted for 80–90% of the yeast population recovered from the plates 1 and 2 days after inoculation with *H. vineae*, whereas *C. zemplinina* had completely disappeared by the second day. *S. cerevisiae* was present at the beginning of fermentation, but at a very low proportion (Figure 2A). Nevertheless, the *S. cerevisiae* population began to increase from the middle to the end of the fermentation, accounting for 60% of the population at the middle of the fermentation and 100% at the end of the fermentation. From this population, up to three different *S. cerevisiae* strains could be identified by interdelta analysis, although QA23 was the most abundant at the end of the fermentation (more than 90%, results not shown). Cross contamination between cellar vats is common in commercial cellars during vintage. Nevertheless, *H. vineae* represented 40% of the yeast population at the middle of the fermentation, which demonstrated the capacity of this yeast to dominate the native microbiota, and a high proportion of its population remained active after the middle of the fermentation. However, this yeast was not able to overcome the initial microbiota in Merlot fermentations inoculated with *H. vineae* (Figure 2C) and exhibited very low recovery on plates at the beginning and middle of the fermentation (≈ 5–7%). Other non-*Saccharomyces* yeasts (*H. uvarum, C. zemplinina*, and *Pichia occidentalis*) outgrew *H. vinae* at these stages. The predominant yeast throughout the fermentation was a non-inoculated *S*. *cerevisiae*, which was recovered from plates and represented 50% of the total population at the beginning of the fermentation.

Macabeo and Merlot fermentations inoculated with *S. cerevisiae* showed similar yeast population patterns (Figures 2B,D). In both cases, the inoculated *S. cerevisiae* was able to rapidly dominate the fermentation because it was the only cultivable yeast recovered throughout the fermentation.

The PCR-DGGE profiles obtained for the DNA extracted directly from the wine during alcoholic fermentation identified the detected yeasts as *S. cerevisiae, H. uvarum, H. vineae*, and *C. zemplinina* (Table 1). *S. cerevisiae* was detected in all fermentations after the first day of inoculation, including in fermentations not inoculated with the commercial *S. cerevisiae*. Nevertheless, the *S. cerevisiae* in these latter fermentations appeared to be a different strain, as evidenced different migration patterns on DGGE gels. *S. cerevisiae* QA23 shows a particularity in PCR-DGGE because it produces a double band, which is not observed in other *S. cerevisiae* strains. All the bands excised from the gel migrating to the same height resulted in at least 99.9% sequence similarity to *S. cerevisiae* type strain. Merlot and Macabeo musts inoculated with *H. vineae* exhibited more yeast diversity at the beginning of the fermentations than musts inoculated with *S. cerevisiae*, and *H. vineae* was detected until the end of these fermentations.

**Table 1:** **The most abundant yeast genera detected by each technique in the different fermentations**.

**Yeast species**		**Macabeo with** ***H. vineae***				**Macabeo with** ***S. cerevisiae***				**Merlot with** ***H. vineae***			**Merlot with** ***S. cerevisiae***		
		**Day 1**	**Day 2**	**Day 4**	**Day 19**	**Day 1**	**Day 2**	**Day 4**	**Day 14**	**Day 1**	**Day 6**	**Day 9**	**Day 1**	**Day 6**	**Day 9**
YPD Culture (%)	*S. cerevisiae*	5,26	<	60,00	100,00	96,30	97,50	100,00	100,00	50,00	77,36	96,61	98,33	88,14	100,00
	*H. vineae*	78,95	90,70	40,00	−	−	−	−	−	5,88	7,55	<	−	<	−
	*H. uvarum*	−	−	−	−	−	−	−	−	26,47	7,55	−	<	−	−
	*C. zemplinina*	15,79	−	−	−	<	<	−	−	−	7,55	<	−	10,17	−
	*P.occidentalis*	−	−	−	−	−	−	−	−	14,71	−	−	−	−	−
HTS (%)	*S. cerevisiae*	<	<	15,20	70,91	97,97	98,23	98,40	98,87	<	85,25	85,93	97,67	96,78	97,20
	*H. vineae*	99,28	95,81	84,39	28,00	<	<	<	<	96,09	5,51	5,53	<	<	<
	*H. uvarum*	<	<	<	<	<	<	<	−	<	7,94	6,77	<	<	<
	*Candida*	<	<	<	<	<	<	<	<	<	<	<	<	<	<
DGGE- PCR	*S. cerevisiae*	−	+	++	++	+	++	++	++	+	++	++	++	++	++
	*H. vineae*	++	++	+	+	−	−	−	++	++	+	+	−	−	−
	*H. uvarum*	−	−	−	−	−	−	−	−	+	+	+	−	−	−
	*C. zemplinina*	+	+	+	+	+	++	++	+	−	−	−	−	−	−

*The YPD-Culture and HTS results are expressed as percentages of the selected yeast specie from the total yeast population. The symbol “<” indicates that the correspondent yeast specie is present but represents less than 5% of the total yeast population. The symbol “−” indicates that the correspondent yeast specie was not detected with this technique. In case of DGGE-PCR, “++” indicates that the band is very strong, “+” indicates that the band is weak and “−” indicates that the band is non-existent*.

**Figure 3:**
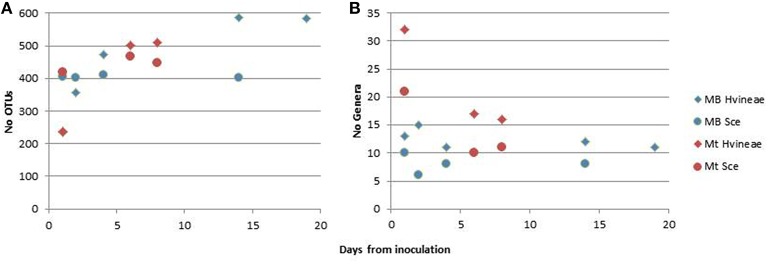
**Number of OTUs (A) and genera (B) detected by HTS after inoculation of ***H. vineae*** (♦) or ***S***. ***cerevisiae*** (O) on Merlot wine (red symbols) or Macabeo wine (blue symbols)**.

A high-throughput sequencing (HTS) approach was also used to assess the fermented wine yeast biodiversity. After the removal of low quality sequences and those failing alignment, 642,105 18S rRNA amplicon sequences were generated from 9 Macabeo and 6 Merlot wine samples. The average number of sequences per sample was 42,807, with an average length of 299 bp, and these sequences clustered into 16,302 operational taxonomic units (OTUs; 97% nucleotide identity). To avoid diversity overestimation, singletons (sequences observed only once) were eliminated, and each sample was rarified to an even depth of 20,000 sequences to avoid biases generated by differences in sequencing depth. The number of different OTUs was then reduced to 634, and 34 genera were identified. Good's coverage index was 99.7% on average, indicating that the global yeast diversity was mostly covered. The numbers of observed OTUs did not differ between Macabeo or Merlot wine samples inoculated with *S. cerevisiae* or *H. vineae* (Figure 3A). However, the number of genera was significantly higher at the beginning of the Merlot fermentation and tended to decrease toward the end of the fermentation, whereas the number of genera in Macabeo fermentation samples was lower than that in Merlot fermentations and relatively constant throughout the fermentation (Figure 3B). Most of the yeast population in all fermentations (97.7% on average) was represented by the inoculated *S. cerevisiae* and *H. vineae* strains (Tables 1, 2), whereas other non-*Saccharomyces*, such as *H. uvarum* and *Zygosaccharomyces*, accounted for only 1.9% of the sequences, and the remaining genera represented less than 0.5% of the sequences (Table 2). Some of the detected fungi were not related to alcoholic fermentation (p.e. *Aerobasidium, Aspergillus, Sporobolomyces*); however, they were mainly detected at the beginning of the fermentation, and their populations quickly decreased or disappeared (Table 2). Interestingly, *Dekkera* was only detected in Merlot samples, and we were able to observe a small but distinct increase during the fermentations with both inocula.

**Table 2:** **Percentage of main genera and species detected by HTS after the inoculation of ***H. vineae*** or ***S. cerevisiae*** on Merlot and Macabeo wines**.

**Days from inoculation**	**Macabeo** ***H.vineae***					**Macabeo** ***S. cerevisiae***				**Merlot** ***H. vineae***			**Merlot** ***S. cerevisiae***		
	**1**	**2**	**4**	**14**	**19**	**1**	**2**	**4**	**14**	**1**	**6**	**8**	**1**	**6**	**8**
*Saccharomyces cerevisiae*	0.47	3.68	15.20	60.32	70.91	97.97	98.23	98.40	98.87	0.44	85.25	85.93	97.67	96.78	97.20
*Hanseniaspora vineae*	99.28	95.81	84.39	38.75	28.00	0.64	0.48	0.57	0.24	96.09	5.51	5.53	0.53	1.69	1.60
*Hanseniaspora uvarum*	0.07	0.07	0.01	<	0.01	0.05	0.02	0.01	<	0.33	7.94	6.77	0.24	0.40	0.15
*Zygosaccharomyces*	0.01	0.05	0.23	0.47	0.60	0.67	0.66	0.52	0.39	0.41	0.49	0.49	0.53	0.51	0.47
*Saccharomyces (others)*	0.01	0.03	0.08	0.31	0.39	0.55	0.54	0.43	0.44	<	0.33	0.36	0.36	0.37	0.36
*Aureobasidium*	<	<	−	0.01	−	−	−	−	−	1.83	0.03	0.02	0.33	0.05	0.05
*Candida*	0.09	0.14	0.03	0.06	0.07	0.11	0.07	0.06	0.05	0.31	0.18	0.46	0.19	0.18	0.12
*Pichia*	0.05	0.17	0.05	<	0.01	−	−	<	−	0.03	0.26	0.26	0.01	0.01	−
*Dekkera*	−	−	−	−	−	−	−	−	−	−	<	0.17	−	−	0.03
*Aspergillus*	−	0.01	−	−	−	−	−	−	−	0.11	<	0.01	0.03	<	−
*Sporobolomyces*	−	−	−	−	−	−	−	−	−	0.08	<	−	0.02	−	−
*Issatchenkia*	0.01	0.02	0.01	0.01	0.01	0.01	−	<	−	0.02	0.01	−	0.01	−	−
*Cryptococcus*	−	<	−	<	−	−	−	−	−	0.07	−	−	0.01	−	−
*Diplodia*	−	−	−	−	−	−	−	−	−	0.07	−	−	<	−	−
*Zygoascus*	−	−	−	−	−	<	−	−	−	0.03	−	−	0.02	0.01	0.01
*Rhizina*	−	−	−	−	−	−	−	−	−	0.04	−	−	0.01	−	−
*Catenulostroma*	−	−	−	−	−	−	−	−	−	0.05	−	−	−	−	−
*Bensingtonia*	−	−	−	0.04	−	−	−	−	−	<	−	−	−	−	−
*Saccharomycodes*	−	−	<	0.02	0.01	−	−	−	<	−	−	−	−	−	0.01
*Scheffersomyces*	−	−	−	−	−	−	−	−	−	0.02	<	<	0.01	−	−
*Wickerhamomyces*	0.01	0.01	<	−	−	−	−	−	−	0.01	−	−	−	−	−
*Cladosporium*	−	−	−	−	−	−	−	−	−	0.01	−	−	0.01	−	−
*Sugiyamaella*	0.01	0.01	<	−	−	−	−	−	−	−	−	−	−	−	−
*Trigonopsis*	0.01	0.01	−	−	<	−	−	−	−	−	−	−	−	−	−
*Lipomyces*	−	−	−	−	−	−	−	−	−	−	0.01	0.01	−	−	−
*Phillipsia*	−	−	−	−	−	−	−	−	−	0.01	−	−	−	−	−
*Wallemia*	0.01	−	−	−	−	<	−	−	−	<	−	−	−	−	−
*Vanderwaltozyma*	−	−	−	−	−	−	−	−	<	−	<	−	−	−	<
*Cochliobolus*	−	−	−	−	−	−	−	−	−	0.01	−	−	−	−	−
*Malassezia*	−	−	−	−	−	<	−	−	−	<	−	<	−	−	−
*Bispora*	−	−	−	−	−	−	−	−	−	<	−	−	<	−	−
*Rhodotorula*	−	−	−	−	−	−	−	−	−	<	−	−	<	−	−
*Metschnikowia*	−	−	−	−	−	−	−	−	−	<	<	−	−	−	−
*Phoma*	−	−	−	−	−	−	−	−	−	<	−	<	−	−	−
*Agaricostilbum*	−	−	−	−	−	−	−	−	−	<	−	<	−	−	−
*Baudoinia*	−	−	−	−	−	−	−	−	−	<	−	−	−	−	−

*The symbol “<” indicates percentages values lower than 0.01 and bigger than 0. The symbol “−“ indicates not detected by HTS*.

### Volatile compound composition

Fifty volatile compounds produced during alcoholic fermentations of natural Macabeo musts inoculated with *H. vineae* and *S. cerevisiae* were identified and quantified in the Macabeo wines. These compounds were classified into 10 groups, (acetates, acids, alcohols, C6 compounds, carbonyl compounds, esters, phenols, lactones, unusual compounds (named here as “rares”) and terpenes). Table 3 shows the mean concentration of the identified volatile compounds. To assess the possible contribution of the different components to the wine aroma, the detection threshold and aroma descriptor reported in the literature are included for each compound.

**Table 3:** **Average concentrations of the two fermentations (± Standard Deviation) in μg/l**.

	***H. vineae***	***S. cerevisiae***	**Odor descriptor**	**Odor threshold (μg/l)**
	**Average SD**	**Average SD**		
**ACETATES**				
Isobutyl acetate	11 ± 1	0 ± 0^*^	N/A	N/A
Isoamyl acetate	222 ± 20	218 ± 93	Banana^a^	30
1,3-Propanediol, diacetate	99 ± 18	160 ± 7	N/A	N/A
Phenethyl acetate	2322 ± 50	47 ± 13^**^	Fruity, honeyed, floral^a^	250
Acetate sum	2653 ± 89	425 ± 100^**^		
**ACIDS**				
Isobutyric acid	74 ± 40	0 ± 0	Acid, fatty^b^	230
Heptanoic acid	231 ± 28	304 ± 35^*^	N/A	N/A
Hexanoic acid	330 ± 35	777 ± 70^*^	Fatty, cheese^a^	420
Octanoic acid	734 ± 12	1757 ± 335	Fatty^a^	500
Decanoic acid	979 ± 31	389 ± 212	Rancid, fat^a^	1000
9-Hexadecenoic acid	479 ± 11	72 ± 57	N/A	N/A
Acids sum	2825 ± 48	3299 ± 708		
**ALCOHOLS**				
Isobutyl alcohol	2388 ± 277	1895 ± 165	Fusel oil, chemical^b^	0,5
1-Butanol	58 ± 9	84 ± 38	Like wine, medicine^a^	150.000
Isoamyl alcohol	36361 ± 4127	61355 ± 5063^*^	Alcoholic, fruity at low concentration^b^	0,3
3-Methyl-1-pentanol	36 ± 1	69 ± 7	Like wine, nail polish^a^	40.000
3-Ethoxy-1-propanol	28 ± 0	108 ± 12	Fruity^b^	
Furfuryl alcohol	12 ± 2	0 ± 0	N/A	N/A
3-(Methylthio), 1-Propanol	321 ± 35	599 ± 281	Sweet, potato^a^	1000
Benzyl alcohol	37 ± 7	0 ± 0	Floral, rose, phenolic, balsamic^a^	200.000
Phenyl ethanol	8099 ± 158	16830 ± 957	Rose, honey^a^	10000
Tyrosol	1855 ± 156	5274 ± 3149	N/A	N/A
Tryptophol	1365 ± 95	0 ± 0^**^	N/A	N/A
Alcohols sum	50557 ± 4276	86214 ± 897^*^		
**C6 COMPOUNDS**				
1-Hexanol	386 ± 7	328 ± 50	Grass just cut^a^	2500
Trans 3-Hexen-1-ol	7 ± 1	129 ± 19	Green^a^	1000
Cis 3-Hexen-1-ol	120 ± 1	0 ± 0^**^	Green, kiwi^a^	400
C6 compounds sum	513 ± 9	457 ± 31		
**CARBONYL COMPOUNDS**				
Acetoin	15 ± 13	56 ± 59	Creamy, butter, fat^b^	0,15
Furfural	9 ± 2	0 ± 0	Fusel alcohol, toasted bread^a^	770
Carbonyl compounds sum	23 ± 16	56 ± 59		
**ESTERS**				
Methyl butyrate	9 ± 4	14 ± 7	N/A	N/A
Ethyl butyrate	62 ± 15	158 ± 38	N/A	N/A
Ethyl hexanoate	81 ± 4	241 ± 24	Green apple^a^	14
Ethyl lactate	8285 ± 378	3071 ± 1915	Strawberry, raspberry^a^	60.000
Ethyl octanoate	79 ± 33	225 ± 9	Sweet, banana, pineapple^a^	500
Ethyl 3-hydroxybutyrate	119 ± 8	52 ± 16	N/A	N/A
Ethyl decanoate	143 ± 46	76 ± 6	Sweet, hazelnut oil^a^	200
Ethyl succinate	1240 ± 47	1775 ± 836	Toffee, coffee^a^	1.000.000
Diethyl malate	88 ± 6	428 ± 165	Green^a^	760.000
Diethyl 2 hydroxy glutarate	233 ± 6	268 ± 67	Grape, green apple, marshmallow^a^	20.000
Diethyl succinate	4012 ± 255	15671 ± 6792	Overripe melon, lavender^a^	100000
Ester sum	14348 ± 509	21979 ± 9334		
**PHENOLS**				
Guaiacol	6 ± 1	0 ± 0	Smoky, hospital^a^	9,5
4-ethylguaiacol	73 ± 66	0 ± 0	Bretty flavors^a^	110
4-vinylguaiacol	33 ± 21	28 ± 14	Clove, curry^a^	40
Phenyl lactate	53 ± 10	128 ± 32	N/A	N/A
Ethyl vanillate	5 ± 0	17 ± 20	N/A	N/A
Acetovainillone	14 ± 5	15 ± 13	N/A	N/A
Phenol sum	183 ± 41	188 ± 11		
**LACTONES**				
Butyrolactone	223 ± 1	251 ± 6	Toasted burned^a^	1000
5-carboethoxy-gamma-butyrolactone	127 ± 7	76 ± 11	N/A	N/A
Lactone sum	350 ± 8	327 ± 17		
**RARES**				
N-acetyl tyramine	2040 ± 11	0 ± 0^**^	N/A	N/A
1H-Indole-3-ethanol, acetate (ester)	1377 ± 8	0 ± 0^**^	N/A	N/A
Rare sum	3417 ± 4	0 ± 0^***^		
**TERPENES**				
Linalool	12 ± 2	28 ± 13	Rose^a^	50
Alpha-terpineol	112 ± 31	0 ± 0	Floral, pine^a^	400
Citronellol	27 ± 6	39 ± 5	Sweet, floral^b^	18
Terpene sum	150 ± 23	67 ± 18		

*Odor descriptor and odor thresholds reported in the literature are included*.

*, **, *** *indicate significance at p < 0.05, p < 0.01, p < 0.001 respectively*.

a *Fariña et al. (2015)*.

b *Boido (2002)*.

**Figure 4:**
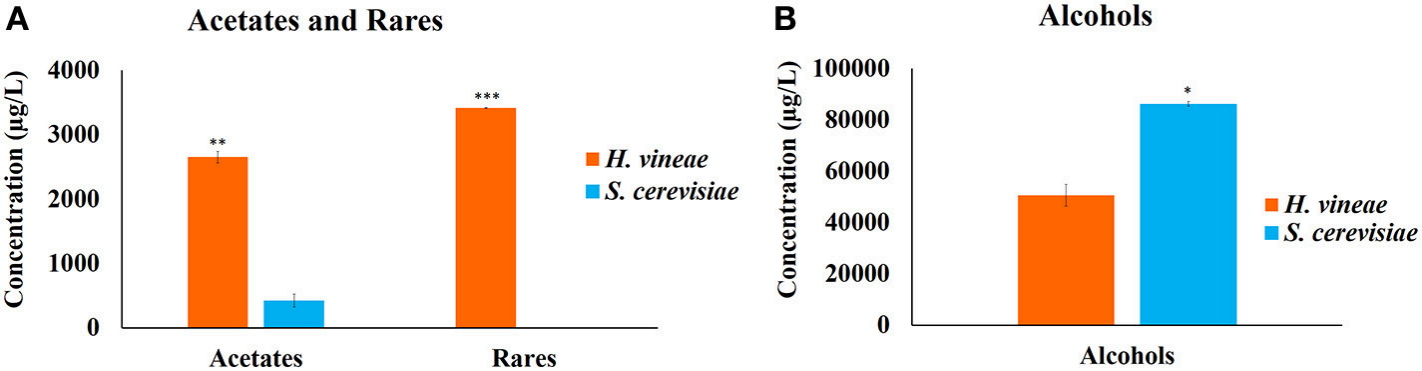
**Sum of compounds with significant differences produced by ***H***. ***vineae*** and ***S. cerevisiae*** (A) acetates and compounds listed as rare (N-acetyltyramine and 1H-indole-3-ethanol acetate ester) (B) Alcohols**. Code: ^*^, ^**^, ^***^ indicate significance at *p* < 0.05, *p* < 0.01, *p* < 0.001, respectively.

Significant differences between yeasts were only observed in three of the 10 groups of compounds (Acetates and rares in Figure 4A and alcohols in Figure 4B).

Both yeasts primarily produced alcohols and esters, and three (isobutanol, isoamyl alcohol, and phenyl ethanol) of the eleven identified alcohols reached the threshold of perception reported in the literature. Of these three alcohols, phenyl ethanol provides good aromas that are described as rose and honey-like. Among the identified esters, ethyl hexanoate reached the threshold of perception and contributes a green apple aroma. The compounds constituting the next most abundant group produced by *H. vineae* are classified as rare and included N-acetyl tyramine and 1H-indole-3-ethanol acetate ester. These compounds were not found in the wine fermented with *S. cerevisiae*.

As shown in Table 3, a total of 7 acids were identified; hexanoic, decanoic and octanoic acid showed the highest concentration, and octanoic acid exceed the odor threshold reported in the literature.

Four acetates were identified, and phenethyl acetate was the most interesting. Specifically, this compound was 50 times more abundant in wines fermented with *H. vineae* than in those fermented by *S. cerevisiae*. This compound endows wine with floral, fruity and honey-like aromas.

Six phenolic compounds were identified, as shown in Table 3. These compounds did not reach the threshold of detection, and their contribution to wine aroma is consequently expected to be insignificant. One of these compounds, 4-ethylguaiacol, is generally attributed to the presence of *Brettanomyces*, although it was identified in wines fermented with *H. vineae*.

Six terpenes were identified, as shown in Table 3. The concentrations of these compounds were lower than the threshold, and they are consequently not expected to contribute to the wine flavor profiles.

### Sensory analysis

To evaluate the ability of *H. vineae* to produce a wine with attributes that differ from those of a wine fermented with *S. cerevisiae*, the produced wines were analyzed with triangle and descriptive tests.

**Figure 5:**
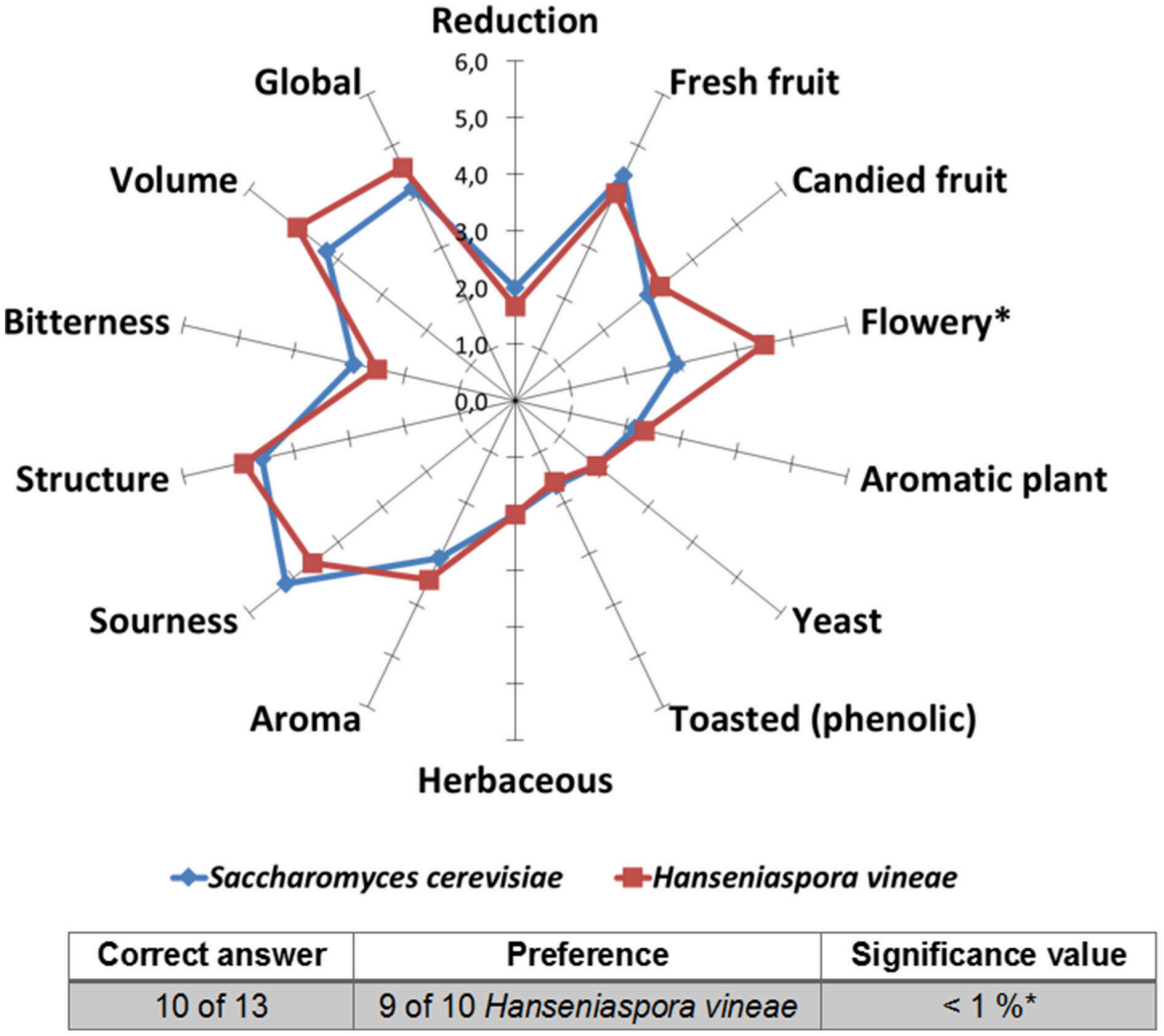
**Results of triangle (table) and descriptive (graphic) test of Macabeo wine fermented with ***H. vineae*** and ***S. cerevisiae*****.

In the triangle test of Macabeo wine (Figure 5), wine-tasters easily distinguished the wine fermented with *H. vineae* from that fermented with *S. cerevisiae*, and the majority selected the wine fermented with *H. vineae* as their preference. In the descriptive test, the wine fermented with *H. vineae* received the best rating. Notably, wine fermented by *H. vineae* showed a significantly stronger flowery aroma profile (*p* = 0.037) than wine produced with *S. cerevisiae*.

The Merlot must could not be evaluated as a consequence of a powerful reduction note that could not be corrected for the tasting.

## Discussion

In this work, we performed semi-industrial fermentations with *H. vineae* and *S. cerevisiae* using Macabeo and Merlot musts in order to evaluate the differences in yeast populations during fermentation and the wines produced. We followed the yeast population dynamics in both grape musts inoculated with *H. vineae* and *S. cerevisiae* by plate culturing on YPD medium, PCR-DGGE with yeast general primers, as well as yeast general primers and the HTS of 18S rRNA gene.

As expected, the fermentation of musts inoculated with *H. vineae* required more time than fermentations inoculated with *S. cerevisiae*, demonstrating the high fermentative ability of this wine yeast with respect to *H. vineae*. However, rapid fermentation might not be a desired characteristic in the production of quality wines, due to flavor lost or high energetic demand for refrigeration (Medina et al., 2013).

The initial yeast diversity of the must was only analyzed after plate culturing. Before the inoculation, Merlot must presented a higher yeast diversity than Macabeo must, as evidenced by up to eleven different yeast species that were identified in the red variety, whereas the Macabeo contained only three species (*C. zemplinina, H. uvarum*, and *T. delbrueckii*). As expected, only non-*Saccharomyces* yeasts were recovered by cultivation from both musts before the inoculation because *S. cerevisiae* is not present in relevant amounts in grapes and is mostly associated with cellar equipment (Pretorius, 2000; Torija et al., 2001; Beltran et al., 2002).

The PCR-DGGE analysis identified *S. cerevisiae* and *H. vineae* as the main yeasts in both the Macabeo and Merlot fermentations. *C. zemplinina* was found only in Merlot, and these results corroborated those observed after the plate culture. Other minor yeast species were not detected by PCR-DGGE, especially if their population densities were below 10^3^–10^4^ CFU/ml or if their abundance was two orders of magnitude lower than that of the main species, as reported in previous studies (Mills et al., 2002; Prakitchaiwattana et al., 2004; Andorrà et al., 2008).

Even if must samples were not included in the HTS approach, this technique clearly detected higher levels of fungal diversity than the other techniques. Specifically, a total of 32 genera with a great diversity of OTUs were identified within each genus. The HTS technique was also able to detect yeast genera not related with fermentation, and some of these yeasts are associated with spoilage (like *Dekkera/Brettanomyces*). Although the proportion of these yeasts was very low, the changes in their proportion throughout the fermentation suggested that they were active and represented a potential risk for the spoilage of the final wine. Thus, the HTS technique confirmed the general trend obtained for the most abundant yeast populations by plate culturing and PCR-DGGE, but it also facilitated the detection and tracking of some minor yeast genera that may significantly impact the quality of the wine.

The culturing, PCR-DGGE and HTS analysis confirmed a decrease in the yeast genera diversity from the beginning to the end of fermentations, and these techniques also consistently indicated that the yeast diversity was higher in Merlot fermentations than in Macabeo fermentations. The low diversity exhibited by Macabeo must before inoculation may be a consequence of its treatment with a vacuum filter. The objective of this treatment was to clean the must and remove solid and colloidal particles, but it also reduced autochthonous yeasts and nutrients in the must. We used this protocol for two reasons: to clean the Macabeo must and to remove colloidal and solid particles and also it was affected by rain and exhibited some spoilage. Thus, we wanted to reduce the autochthonous yeast population because we planned to inoculate the must with *H. vineae*. We achieved these objectives. Furthermore, the Merlot was selectively handpicked in order to obtain the healthiest bunches of grapes. The results from plate culturing, PCR-DGGE and HTS indicate that *H. vineae* was able to overcome the autochthonous microbiota in the Macabeo must, constituting a high proportion of the yeast population until the middle of the fermentation and showing good fermentative capacity. However, *H. vineae* represented a very low proportion of the yeast population in Merlot must after the inoculation. However, after the inoculation (day 1), the percentages of the identified yeasts were different based on the method of estimation, being the population of *H. vineae* hardly recovered on plates. *S. cerevisiae* was the most abundant yeast recovered from plates, whereas it was present at much lower levels in all culture-independent methods (HTS and DGGE). This observation could be related to the well-reported interaction between *S. cerevisiae* and non-*Saccharomyces* yeasts during wine fermentation: non-*Saccharomyces* yeasts are quickly displaced by *S. cerevisiae*, which might kill or at least result in viable but not cultivable (VBNC) statuses, as indicated in several recent reports (Millet and Lonvaud-Funel, 2000; Pérez-Nevado et al., 2006; Andorrà et al., 2010, 2011; Wang et al., 2015). However, we should emphasize that these culture-independent techniques also detect DNA from dead cells, which could also be the case. At later fermentation time points, all methods again produced coincident results and identified *S. cerevisiae* as the main population. Interestingly, the dominant *S. cerevisiae* was not the inoculated strain, suggesting that a cellar-resident strain took over. Furthermore, Merlot grapes are among the latest in the harvest in this cellar, and, thus, the environmental contamination of the cellar is already high. The *S. cerevisiae* population began to increase and became the dominant species according to HTS and produced the most intense band profile of DGGE, and this unique yeast was recovered at the end of the fermentation.

The final wine obtained by fermenting Macabeo must with *H. vineae* was preferred over the wine fermented with *S. cerevisiae* for its notable fruity and flowery aroma. This result corroborates those of studies that performed mixed fermentations with *H. vineae* and obtained high amounts of an acetate ester, phenethyl acetate, which is responsible of the fruity and flowery aroma of wine (Viana et al., 2009, 2011). The chemical analysis revealed that wines inoculated with *H. vineae* contained 50 times more phenethyl acetate than wines inoculated with *S. cerevisiae*, which explains the results of our sensory analysis and agrees with previous observations (Medina et al., 2013).

The production of N-acetyltyramine and 1H-indole-3ethanol acetate ester also differed. These compounds were abundant in wines inoculated with *H. vineae* and could not be detected in wines fermented with *S. cerevisiae*. These compounds could be derived from tyrosol, and this hypothesis is supported by the high concentrations of tyrosol in wines inoculated with *S. cerevisiae*. This difference could be explained by the production of unusual compounds from tyrosol in wines inoculated with *H. vineae*. However, aromatic descriptors associated with these compounds have not yet been reported.

## Conclusion

Interest in the use of non-*Saccharomyces* yeasts in winemaking has been increasing. *H. vineae* is an apiculate non-*Saccharomyces* yeast that has demonstrated a good fermentative rate in Macabeo must and resulted in more flowery wines, which is likely related to the higher production of phenylethyl acetate. However, the need for inoculation with *S. cerevisiae* must be emphasized because *H. vineae* is unable to finish the alcoholic fermentation. We did not use a *S. cerevisiae* strain in the inoculations with *H. vineae*, and the end of fermentation was consequently improperly controlled. Furthermore, the use of this yeast requires very healthy grape musts and is not recommended to use with grapes with a high and diverse yeast population or red musts, in which maceration with skins may be a significant source of yeast. In addition, the present study shows that the HTS technique detected not only the most abundant yeast populations obtained by plate culturing and PCR-DGGE but also some minor yeast genera that may significantly affect the quality of the wine.

## Author contributions

JL: Performing the experiments, Design of experiments, Writing of the manuscript, Discussion of results, Performing Next Generation Sequencing, Analysis of results. VM: Performing the experiments, Design of experiments, Analysis of results, Discussion of results, Writing of the manuscript. MP: Performing Next Generation Sequencing, Analysis of results, Writing of the manuscript. FC: Design of experiments, Analysis of results, Discussion of results. GB: Design of experiments, Analysis of results, Discussion of results. AM: Design of experiments, Discussion of results, Analysis of results, Writing of the manuscript.

### Conflict of interest statement

The authors declare that the research was conducted in the absence of any commercial or financial relationships that could be construed as a potential conflict of interest.
